# Green Perspectives for Public Health: A Narrative Review on the Physiological Effects of Experiencing Outdoor Nature

**DOI:** 10.3390/ijerph110505445

**Published:** 2014-05-19

**Authors:** Daniela Haluza, Regina Schönbauer, Renate Cervinka

**Affiliations:** Institute of Environmental Health, Center for Public Health, Medical University of Vienna, Kinderspitalgasse 15, A-1090 Vienna, Austria; E-Mails: regina_schoenbauer@gmx.at (R.S.); renate.cervnika@meduniwien.ac.at (R.C.)

**Keywords:** blood pressure, brain activity, cardiovascular activity, cortisol, endocrine system, forest, heart rate, immune function, outdoor nature, physiology

## Abstract

Natural environments offer a high potential for human well-being, restoration and stress recovery in terms of allostatic load. A growing body of literature is investigating psychological and physiological health benefits of contact with Nature. So far, a synthesis of physiological health outcomes of direct outdoor nature experiences and its potential for improving Public Health is missing. We were interested in summarizing the outcomes of studies that investigated physiological outcomes of experiencing Nature measuring at least one physiological parameter during the last two decades. Studies on effects of indoor or simulated Nature exposure via videos or photos, animal contact, and wood as building material were excluded from further analysis. As an online literature research delivered heterogeneous data inappropriate for quantitative synthesis approaches, we descriptively summarized and narratively synthesized studies. The procedure started with 1,187 titles. Research articles in English language published in international peer-reviewed journals that investigated the effects of natural outdoor environments on humans by were included. We identified 17 relevant articles reporting on effects of Nature by measuring 20 different physiological parameters. We assigned these parameters to one of the four body systems brain activity, cardiovascular system, endocrine system, and immune function. These studies reported mainly direct and positive effects, however, our analyses revealed heterogeneous outcomes regarding significance of results. Most of the studies were conducted in Japan, based on quite small samples, predominantly with male students as participants in a cross-sectional design. In general, our narrative review provided an ambiguous illustration of the effects outdoor nature exerted on physiological parameters. However, the majority of studies reported significant positive effects. A harmonizing effect of Nature, especially on physiological stress reactions, was found across all body systems. From a Public Health perspective, interdisciplinary work on utilizing benefits of Nature regarding health promotion, disease prevention, and nature-based therapy should be optimized in order to eventually diminish given methodological limitations from mono-disciplinary studies.

## 1. Background

Spending leisure time in a green environment has positive effects on prevalence of diseases and mortality rates as well as on perceived mental and general health [1,2,3,4,5,6]. Furthermore, Nature and green spaces offer an inexpensive resource for enhancing physical activity and thus reducing stress-associated and life style-related disorders such as the burnout syndrome, obesity, and cardiovascular diseases [7,8]. Closely meshed interdisciplinary collaboration and communication is a prerequisite to evaluate diverse nature-based interventions e.g., Horticultural Therapy [9,10,11]. 

Evidence-based data on health-enhancing effects of Nature is of interest for various stakeholders including medical professionals, landscape architects, urban designers, and economic experts. To provide a sound basis of knowledge, which is essential for interdisciplinary approaches, several papers offer an overview on research of the health effects of Nature [12,13,14]. The majority of previous research has focused on psychological variables like concentration [15], affects/emotions [16], well-being [17], and mood [18]. However, besides psychological outcomes, there is growing interest in investigating complex health-prompting effects of Nature by measuring physiological indicators [9]. 

The concept of allostatic load, coined by McEwen and Stellar [19], characterizes stress responses and adaptive processes, and thus, can be quantified by the amount of stress-mediating physiological agents [20,21]. In contrast to reports on psychological findings, studies on the impact of contact to outdoor Nature on allostatic load by measuring physiological parameters are only roughly outlined in the respective literature. Also, investigations of these physiological outcomes do not seem to find as consistent results as psychological factors [14]. 

According to Maas and Verheij, general practitioners do not integrate Nature in counseling on health promotion and disease prevention [22]. To “prescribe” natural outdoor environments as an efficacious remedy to their patients, medical professionals need to have access to specific evidence-based knowledge on various markedly favourable effects of outdoor Nature on physiological health outcomes [23,24]. A synopsis of hitherto conducted related studies, their reported findings, and the applied methods is favourable for everyday doctor-patient-communication and shaping future Public Health-related research efforts. 

With respect to Public Health aspects, the present paper aimed at summarizing and reviewing existing literature on physiological effects of experiencing Nature in order to provide a summary of knowledge on given evidence and illuminate current trends mirrored in the past two decades of research. Basically, three research foci guided our analyses: (i) Which physiological parameters are used for investigating health benefits of Nature? (ii) Which of the reported findings reach statistical significance? (iii) Which research design characterizes the respective studies?

## 2. Method

### Design and Methods

In the initial planning phase of this study reviewing the current scientific knowledge regarding physiological responses associated with Nature contact, a preliminary literature search retrieved a limited number of empirical studies, employing mainly cross-sectional and quite heterogeneous designs. Due to the lack of randomised controlled trails and longitudinal research, we assumed that systematic reviews and meta-analysis in order to generate evidence-bases conclusions are not yet warranted in this research field [25]. In general, a systematic review investigates a clearly defined topic or question following a clear search protocols. In contrast, we intended to provide an overview of published data by means of a narrative review approach using an evidence evaluation system to rank the quality and strength of individual studies and to derive recommendations based on the consistency and strength of the underlying evidence [26].

Thus, we conducted a four step approach aimed at retrieving research articles published in the twenty years from January 1991 to January 2012 to capture respective research trends during a time period of the last two decades. First, we used systematic snowball sampling adapted from the literature for collecting respective publications [27]. We applied prospective snowballing to identify a set of five papers, which we considered to be key review articles dealing with restorative effects of benefits of Nature on health and well-being [10,14,28,29,30]. Based on these articles, we defined the research focus, inclusion as well as exclusion criteria for the retrospectively performed literature search. The inclusion criteria were as follows:

- Empirical study
- Publication date between January 1991 and January 2012 (20 year period)
- Published in peer-reviewed scientific journal
- Research conducted internationally
- Full-text article available in English language
- Study subjects were adults
- Investigation of effects of natural outdoor environments including urban green by measuring at least one physiological parameter
- (Statistical) inter-group comparison of effects

Contrarily, research articles on effects of simulated/indoor Nature, animal contact, and wood as building material were excluded from the analysis.

Second, we merged the bibliographic references of these five reviews comprising 1,187 titles and abstracts that were screened by three independent reviewers in a consensus-orientated process. According to the defined inclusion criteria, four papers matched these inclusion criteria [31,32,33,34]. 

Third, bibliographic references of these four articles as well as the related full-text articles were screened for additional relevant studies, identifying eight further papers matching the inclusion criteria [15,35,36,37,38,39,40,41].

Finally, as a forth step, we extracted a list of key words from these hitherto retrieved 12 articles and used all possible two-word combinations and appreviations (*): “Physiologic *”, “natur *”, “green”, “outdoor”, “restorati *”, and “stress”. Manual searching through the bibliographic references of selected articles supplemented the electronic enquiry in four electronic databases (Central, Medline, Embase, and Social Sciences Citation Indexes). This online search revealed five additional publications [42,43,44,45,46]. 

In total, our literature searches yielded 17 articles available in full-text for subsequent eligibility assessment according to the inclusion/exclusion criteria, methodological consistency, and research outcome. Using a narrative synthesis, findings were summarized, tabulated and synthesized in regard of the three research foci, *i.e.*, physiological parameters, significance of effects, and methodological characteristics. 

Concerning physiological parameters, we assigned identified parameters to one of the four body systems: (i) brain activity, (ii) cardiovascular system, (iii) endocrine system, and (iv) immune function. These classification categories included the most common physiological characteristics observed in research practice and have already been partly used by other authors [14]. 

Further, we referred to study results as “significant positive” if all of the comparisons in the respective study showed positive effects of nature (significant at 5% level). The label “mixed results” indicated that only some comparisons showed significant positive effects of Nature. Furthermore, “insignificant” labelled reported comparisons with statistically insignificant effects. To assess methodological study characteristics, we used a data extraction sheet to gather publication-specific data including variables such as year and author(s), country of origin, study design (sample size, sex, and population), environmental settings and time frame over which exposure took place.

## 3. Results

From the included research articles, we derived 20 different physiological parameters reflecting effects of exposure to outdoor nature [15,31,32,33,34,35,36,37,38,39,40,41,42,43,44,45,46].

### 3.1. Physiological Parameters used for Investigating Health Benefits of Nature in the Last Two Decades

The following section summarizes information regarding year of publication and specific physiological parameters by the four body systems:

1. Brain activity. Park and co-workers reported on the influence of staying in a forest on prefrontal cerebral activity [34].
2. Cardiovascular activity. Twelve research studies published between 1998 and 2011 focused on cardiovascular effects, including blood pressure [15,31,33,34,35,38,39,40,41,43,46], heart rate [15,34,36,38,39,40,41,42,46], and heart rate variability [15,34,36,38,39,40,41,42,46]
3. Endocrine system. In total, nine endocrine parameters were investigated—one investigation was published in 1998 [35], whereas twelve studies have been published after the year 2002: adiponectin [43], adrenaline [37,43], blood glucose [35], cortisol [33,34,36,38,39,40,42,44,45], dehydroepiandrosterone sulphate [43], dopamine [43,47], glycated haemoglobin A_1c_ [35], noradrenaline [37,43], and salivary amylase [32].
4. Immune function. Two recently published studies examined Nature’s effects on parameters of immune function in body fluids of study subjects. Li *et al.* analyzed CD3^+^ cells, granulysin, granzymes A/B-expressing cells, natural killer cells, perforin, and white blood cell count in female participants, whereas Tsunetsugu and colleagues reported on immunoglobulin A concentrations in saliva of male subjects [33,37].

During the last decades, cardiovascular parameters were scope of continuous research efforts. In our analysis, we found a strong emphasis on cardiovascular factors compared to other physiological parameters. Within this category, eleven out of twelve studies measured participants blood pressure, nine studies measured heart rate, and additionally, half of them reported on heart rate variability. In contrast to effects on cardiovascular parameters, especially blood pressure, most endocrine parameters have been investigated only in a few papers. An exception was cortisol, measured in nine recent studies. In sum, a strong emphasis on stress indicators is evident in scientific research of the last twenty years.

### 3.2. Significance of Differences in Health Effects

**Figure 1:**
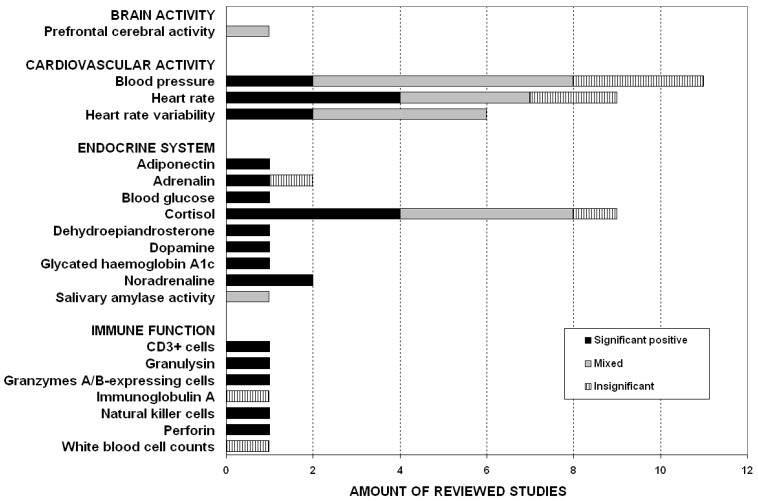
Study outcomes stratified by amount of studies (total n = 17) and physiological parameters. Significant positive (black bars), mixed (grey bars), and insignificant (black and white bars) results are depicted.

Figure 1 displays the significance of results reported in the reviewed studies, following the systematic of the four body systems:

1. Brain activity. A single publication on nature’s effect on prefrontal cerebral activity conducted by Park *et al.* revealed mixed results [34].
2. Cardiovascular activity. Two studies analyzing blood pressure found significant positive effects of outdoor nature environments [35,40], six studies showed mixed results [31,33,38,41,43,46], and finally, three studies found insignificant effects [15,39,42]. Concerning heart rate, four studies found significant positive effects [39,40,42,46], three studies reported mixed results [33,38,41], whereas two studies revealed insignificant results [15,36]. Moreover, heart rate variability was investigated in six research articles: Two studies revealed significant positive effects [40,41], however, four articles reported mixed effects of nature [33,36,39,42].
3. Endocrine system. Taking a closer look on endocrine functions, Li and co-workers reported on significantly increased serum adiponectin levels after contact with a forest environment [43]. Nature’s effect on adrenaline levels was investigated in two research articles. Whereas Li *et al.* found significant positive effects [37], Li *et al.* reported insignificant results [43]. However, contact with Nature significantly reduced participants` blood glucose levels [35]. Cortisol levels decrease was either reported as significant positive [38,40,44,45], mixed [33,34,39,42], or insignificant [34]. Significant reduction in dehydroepiandrosterone sulfate (DHEA-S) and dopamine levels were reported by Li and co-workers [43]. Ohtsuka and colleagues revealed positive significant decrease in glycated haemoglobin A_1c_ concentrations [35]. Two investigations conducted by Li *et al.* revealed significant positive effects of nature concerning reduction of noradrenaline concentration [37,43]. Yamaguchi *et al.* found mixed effects on a decrease in salivary amylase activity [32].
4. Immune function. Outdoor nature exposure showed significant positive effects on parameters characterizing immune function-related responses including granulysin, perforin as well as CD_3_^+^-, NK-, and granzymes A/B-expressing cells, [37]. On the other hand, insignificant results were found in regard of immunoglobulin A levels [38] and white blood cell count [37].

Concluding from results presented in Figure 1, significant positive outcomes (n = 25) dominate over mixed (19) and insignificant findings (n = 9). However, these results should be interpreted with care, as the classification of results as significant positive, mixed, or insignificant resulted from summarising series of comparisons conducted within the respective studies (as described in the Method section). For example, the assignment as significant positive indicates that all statistical comparisons reported in the respective study delivered significant positive results. Though, this classification doesn’t provide information about the number of conducted comparisons within the respective study, because these absolute numbers were not reported in the papers. Accordingly, this limitation also applies to the label “insignificant”. Furthermore, the classification as “mixed” indicates at least one result that reached statistical significance, but does not picture the number of positive and insignificant comparisons within the respective study. 

### 3.3. Methodological Characteristics of Reviewed Literature

Study characteristics stratified by publication date (Table 1) and methodological details (Table 2) are tabular-wise assembled to show sample characteristics, exposure-related study design, and investigated settings. The comprehensive reference list additionally provides an overview of respective number of studies and respective authors. 

**Table 1:** Description of study characteristics of reviewed article (n = 17) stratified by publication date.

Study Information			Study Sample			Study Design		Reference
Year	Authors	Country	Size	Sex	Population	Exposure	Setting	
1998	Ohtsuka *et al.*	Japan	87	m/w	Diabetic patients	30 or 60 min	Longitudinal. Forest, sitting/walking	[35]
2002	Rodiek	USA	17	w	Residents of nursing facility	<150 min	Outdoor (garden) *vs.* indoors	[44]
2003	Hartig *et al.*	USA	112	m/w	Students	60 min	Nature reserve *vs.* urban area, sitting/walking	[31]
2005	Ottoson & Grahn	Sweden	15	m/w	Residents of nursing facility	60 min	Outdoors (garden) or indoors, resting	[15]
2006	Gathright *et al.*	Japan	11	m/w	Inexperienced climbers	unknown	living tree or concrete tower in forest, climbing	[36]
2006	Yamaguchi *et al.*	Japan	10	m	Students	20 min	Cross-over trials forest *vs.* urban area, sitting/walking	[32]
2007	Park *et al.*	Japan	12	m	Students	20 min	Cross-over trials forest *vs.* urban area, sitting/walking	[34]
2007	Tsunetsugu *et al.*	Japan	12	m	Students	15 min	Cross-over trials forest *vs.* urban area, sitting/walking	[33]
2008	Li *et al.*	Japan	13	w	Nurses	120 min	Three different forest fields, walking	[37]
2008	Park *et al.*	Japan	12	m	Students	15 min	Cross-over trials forest *vs.* urban area, sitting/walking	[39]
2009	Lee *et al.*	Japan	12	m	Students	30 min	Cross-over trials forest *vs.* urban area	[38]
2009	Park *et al.*	Japan	12	m	Students	15 min	Cross-over trials forest *vs.* urban area, sitting/walking	[41]
2010	Kjellgren & Buhrkall	Sweden	18	m/w	Stressed/burn-out patients	30 min	Simulated *vs.* real nature, sitting	[46]
2010	Park *et al.*	Japan	12	m	Students	30 min	Cross-over trials forest *vs.* urban area, sitting/walking	[40]
2011	Lee *et al.*	Japan	12	m	Students	15 min	Cross-over trials forest *vs.* urban area, sitting	[42]
2011	Li *et al.*	Japan	16	m	Healthy males	120 min	Urban *vs.* forest, walking (morning and afternoon)	[43]
2011	Van den Berg & Custers	The Netherlands	30	m/w	Allotment gardeners	30 min	Performing stressful task, outdoors *vs.* indoors	[45]

**Table 2:** Description of study characteristics of reviewed article (n = 17) stratified by methodological details.

Category	Study Characteristics	References
**Study sample**	**Size**	
	n = 10–18	[15,32,33,34,36,37,38,39,40,41,42,43,44,46]
	n = 30	[45]
	n = 87	[35]
	n = 112	[31]
	**Sex**	
	Males	[32,33,34,38,39,40,41,42,43]
	Females	[37,44]
	Mixed	[15,31,35,36,45,46]
	**Participants**	
	Students	[31,32,33,34,38,39,40,41,42]
	Elderly people	[15,44]
	Diabetic patients	[35]
	Stress/Burnout syndrome patients	[46]
	Climbers	[36]
	Nurses	[37]
	Healthy men	[43]
	Allotment gardeners	[45]
**Exposure**	**Cross-sectional study design**	
	15–20 min	[32,33,34,39,41,42]
	30 min	[38,40,45,46]
	60 min	[15,31]
	120 min	[37,43]
	<150 min	[44]
	Unspecified	[36]
	**Longitudinal study design**	
	9 × 30 min or 60 min over 6 years	[35]
**Setting**	**Environment**	
	Urban *vs.* nature	[31,32,33,34,38,39,40,41,42,43]
	Outdoor *vs.* indoor	[15,44,45]
	Nature	[35,36,37,46]
	**Landscape**	
	Forest	[32,33,34,38,39,40,41,42,43,46]
	Garden	[15,44,45]
	Wildlife reserve	[31]
	**Geographical area**	
	Japan	[32,33,34,38,39,40,41,42,43]
	Europe	[15,45,46]
	USA	[31,44]

Although most of the studies had student participants, there was also research on various other groups e.g., elderly people, allotment gardeners, and diabetic patients. The majority of the studies compared urban with Nature environments; less common were comparisons of indoor and outdoor environments or simply studying effects of Nature without employing a control group design. We identified the forest as the most investigated natural outdoor environment, but there were also some studies investigating the effects of gardens. One single study reported on walking in a wildlife reserve. 

Although we also retrieved articles presenting research conducted in Europe and the USA, the core of analyzed studies was built by eight Japanese papers focusing on “Shinrin-Yoku”. Shinrin-Yoku means “forest bathing” and is defined as “making contact with Nature and taking in the atmosphere of the forest” [32,33,34,38,39,40,41,42]. These Shinrin-Yoku studies employed comparable study designs: small samples (n = 10–12) of male students were sent to urban or forest environments, respectively. Physiological and psychological parameters were measured before and after 15–20 min walking or sitting. Summing up results presented in Table 1 and Table 2, the analyzed publications predominantly investigated quite small samples of male students participating in cross-sectional designed studies conducted in Japan, reflecting the importance of Shinrin-Yoku in this region.

## 4. Discussion

To the best of our knowledge, narrative reviews with a restricted focus on physiological outcomes and research design of research articles on effects of outdoor Nature have been lacking so far. Thus, our article including 17 articles [15,31,32,33,34,35,36,37,38,39,40,41,42,43,44,45,46] expands and updates the findings of earlier reviews combining literature investigating both physiological and psychological outcomes of Nature [14]. As this narrative review integrates findings from studies in which a broad array of methods were applied, pooling was not feasible [25], so, we collected data and synthesized findings guided by a snowball sampling approach. 

Even though we focussed on analyzing original articles on physiological effects, 14 out of 17 studies additionally reported on psychological aspects such as mood and emotions [31,33,34,36,37,38,39,40,41,42,44,45,46]. Furthermore, two studies reported on attention [15,31]. Thirteen out of 14 articles found at least one significant positive effect of Nature on these various psychological parameters [15,31,33,34,36,37,38,39,40,41,42,44,45,46]. 

This narrative review included articles that studied a considerable number of physiological parameters belonging to the cardiovascular and endocrine system. We reason that the predominance of non-invasive measurements, e.g., analyzing saliva components, might be due to economical reasons of field research (being quite cheap, easy obtainable, and reproducible in outdoor settings). In the same verve, a steadily growing number of related articles published since this review’s cut off search date, *i.e.*, January 2012 also investigated Nature effects by means of measuring heart rate, blood pressure as well as levels of salivary cortisol and other stress markers [48,49,50,51]. 

Short-term restorative effects of outdoor Nature could be found for almost all measured physiological parameters. However, we observed contradictory outcomes for some of these measures, as it was the case for all cardiovascular activity-related parameters as well as the endocrine stress hormones adrenaline and cortisol, shown in Figure 1. If significant findings have been reported, they indicated beneficial effects associated with a decrease of stress, suggesting that Nature is beneficial for human well being. This finding is in line with a recent meta-analysis on 25 studies dealing with physiological and psychological outcomes of activities in natural and synthetic environments [14]. Herein, Bowler *et al.* reported that pooled effect sizes for physiological outcomes were slightly positive, but not significant. 

Factors influencing heterogeneity of outcomes could include low assessment quality, in particular due to participant factors (socio-demographic or disease status), outdoor settings (weather features), type of intervention (components, intensity, timing), and appropriateness of the respective control group and statistical power (small or inadequate sample sizes). 

Besides this, analysis of methodological details uncovered several aspects for contradictory outcomes of these studies and could serve as possible explanations for inconsistency of effect significance of Nature on specific parameters. In general, physiological parameters measured in field experiments may be affected by manifold factors including the study environment, presence of other participants and investigators, expectations and fears concerning the experiment, as well as physical and mental condition of participants. More specifically, the measuring procedure itself (e.g., collecting of blood or saliva samples) might have a high potential to be a stress factor for study subjects. Especially with regard to allostatic load, parameters with physiological circadian or cyclical variations (e.g., cortisol, reaching peak bodily concentrations in the morning) should be considered when interpreting nature’s effects [52]. Thinking a step ahead, Lee *et al.* argued that the influence of affective forecasting may partly explain differences in cortisol measures in the morning before the experiment [42]. 

As a vast majority of articles reported on solely male study subjects, our narrative review does not allow conclusions on possible gender influences of Nature’s health effects [32,33,34,38,39,40,41,42,43]. A possible reason might be that for specific study designs hormonal fluctuations in females would have to be considered [42]. However, gender-specific research is an essential issue for all aspects of Public Health and thus, should not be neglected [53]. 

Possibly, sample size could also explain inconsistencies in the findings. As physiological parameters are affected by many different influences, it is conceivable that effects of Nature are rather small. To reliably detect small effects, sample sizes of at least 200 participants are recommended [54]. As shown in Table 1 and Table 2, most sample sizes of the analyzed studies were substantially smaller, the largest sample comprised 112 participants [31]. Regarding statistical data analysis, Univariate designs should be complemented by multivariate approaches, investigating Nature’s effect on patterns of physiological reactions or a group of parameters, for which we would recommend to utilize the body systems scheme presented in this paper.

Our literature search identified only a limited number of respective research performed in Sweden and The Netherlands, *i.e.*, countries of Northern Europe (n = 3) [15,45,46] and in the USA (n = 2) [31,44]. We assume that an increase of international research efforts could help to assess the transferability and generalizability of these local results to other geographical areas worldwide [55]. 

The majority of studies reviewed were conducted in Japan (n = 12, Table 1 and Table 2) [32,33,34,35,36,38,39,40,41,42,43,56], maybe due to an unspecified, but pronounced scientific and sociocultural interest in Shinrin-Yoku. According to Tsunetsugu *et al.*, public attention as well as research endeavours to quantify health effects of Shinrin-Yoku was increased during the last decade [57]. For example, Li and colleagues investigated NK cell activity and expression of anti-cancer proteins in forest bathing participants [37]. Moreover, several investigations using comparable study settings and published by the same research group were not included in this narrative review because these articles were not retrieved by the employed online search protocol [56,58,59]. 

In these Shinrin-Yoku studies, beneficial physiological effects of natural environments were often already detectable before experimental exposure, concluding that physiological parameters might adapt promptly when people anticipate contact with natural outdoor environments. Longitudinal studies in this research field are still rare and the only respective study found in our literature search was already published in the year 1998 [35]. Hence, we agree with Hartig *et al.* who claimed a lack of research on long-term as well as cumulative effects of different natural environments [31]. 

There is still a lack of consolidating health policies with the already existing knowledge of favourable health effects of outdoor nature environments. Therefore, we propose to establish the expression “Green Public Health”, loosely following the catchy term “Vitamin G”, suggested by Groenewegen and coworkers to raise awareness for utilizing all kinds of natural resources for Public Health promotion [60]. 

Recently, a growing body of literature focused on the relationship between natural outdoor environments and its possible implications for Green Public Health perspectives [1,2,3,5,14,60,61,62]. Systematic review findings have been shown to be useful in assisting experts and stakeholders in the health care sector in effective decision making [63,64,65]. Therefore, the major achievement of our present review could be seen in enhancing the societal impact of scientific knowledge on Nature’s effect on human health. To emphasize on the urgent need to promote Green Public Health, we consolidated the outcomes in a review article addressing health professionals and related stakeholders [66]. The most evident finding was the short-term stress-reducing potential of natural outdoor environments. With regard to the growing prevalence of the burnout syndrome and other stress-related diseases increasing the allostatic loads of individuals, the data of the current scientific literature suggests to use the resource nature for primary, secondary, and also tertiary stress prevention [67,68]. 

Additionally, from a Green Public Health perspective and in synopsis with the presented results and limitations of previously conducted studies, future research on restoration should place more emphasize on physiological health effects. A more consistent body of evidence might stimulate Public Health and healthcare professionals to count on the health-promoting power of natural environments. It was beyond the scope of our review to include research articles on natural indoor environments or simulated nature, which would be also an interesting and important Green Public Health topic and should clearly be subject to a separate review article. 

As with other types of research, several limitations of this narrative review have to be taken into consideration. Possible occurrence of reporting or publication bias is an important aspect when drawing conclusions from a scientific literature review [69]. Although some physiological parameters might have been investigated, results have not yet been shared with the scientific community due to various reasons including insignificance or inconsistency of outcomes. 

According to the considerable amount of retrieved studies, we assume that southeast Asia/Japan is the present centre of scientific research on health effects of natural environments. Due to absence of translation resources, only articles published in English in peer-reviewed journals and reports with full-text access were included, which may have introduces language, cultural, and/or publication bias. 

We summarised findings on different parameters regarding significance graphically (Figure 1), as it was not appropriate to pool these results. Further, as only a limited number of studies reported the same outcomes, it was not warranted to statistically explore factors influencing heterogeneity (*i.e.*, significant positive, mixed, and insignificant) of study findings. In addition, a lack of detailed methods description and information on results throughout most of the reviewed articles hampered providing evidence for how different interventions influenced health outcomes. 

However, as a major strength of this narrative synthesis, summarized presentation of key findings from each study could facilitate interdisciplinary and transdisciplinary communication concerning beneficial health effects of Nature among stakeholders. The conclusions drawn herein are strengthened by the fact that only peer-reviewed empirical studies identified by searching commonly used electronic literature databases were considered. We strived to compensate the low number of available articles by using a four-step approach and a quite wide-ranging collection of search terms. Moreover, a similar evaluation also only included 15 papers [70]. 

## 5. Conclusion

The findings of this narrative review demonstrate a tendency towards a health-promoting and disease-preventing potential of contact with natural outdoor environments compared to urban settings in terms of a decrease of allostatic load. Availability of evidence-based knowledge on health promoting effects of contact with nature could influence future Green Public Health policies. Thus, the data suggest to improve the methodological quality of research on Nature`s influence on physiological measures by using large samples being representative in terms of demographic characteristics and using longitudinal study designs. Also, further research needs to control for potential confounders and analyze relevant moderating and mediating mechanisms of investigated health effects. 
